# Upregulated ankyrin repeat-rich membrane spanning protein contributes to tumour progression in cutaneous melanoma

**DOI:** 10.1038/bjc.2011.18

**Published:** 2011-02-22

**Authors:** Y H Liao, S M Hsu, H L Yang, M S Tsai, P H Huang

**Affiliations:** 1 Department of Dermatology, National Taiwan University Hospital, College of Medicine, National Taiwan University, No. 7, Chung-Shan South Road, Taipei 100, Taiwan; 2 Department of Pathology, National Taiwan University Hospital, College of Medicine, National Taiwan University, No. 7, Chung-Shan South Road, Taipei 100, Taiwan; 3 Graduate Institute of Pathology, College of Medicine, National Taiwan University, No. 1, Sec. 1, Ren-Ai Road, Taipei 100, Taiwan

**Keywords:** ARMS, cutaneous melanoma, tumour thickness, melanoma progression, MEK/ERK

## Abstract

**Background:**

We have previously demonstrated that overexpression of ankyrin repeat-rich membrane spanning (ARMS) protein facilitates melanoma formation via conferring apoptotic resistance. This study aims to investigate whether ARMS contributes to melanoma progression.

**Method:**

Using immunohistochemistry, we graded the expression level of ARMS in 54 cases of primary melanoma and 46 cases of metastatic melanoma. The immunointensity of ARMS was statistically correlated with individual clinicopathological characteristics. By RNA interference, stable melanoma cell clones with ARMS-knockdown were constructed, and were used for *in vitro* scratch wound, transwell invasion assays, and *in vivo* lung metastasis experiment.

**Results:**

Stronger immunointensity of ARMS was observed mostly in melanomas with Breslow tumour thickness >1.0 mm (Fisher's exact test, *P*=0.002) or with nodal metastasis (Fisher's exact test, *P*=0.026), and was correlated with a worse overall survival in melanoma patients (log-rank test, *P*=0.04). Depletion of ARMS inhibited migration, invasion, and metastatic potential of melanoma cells *in vitro* and *in vivo*. Moreover, ARMS mediated melanoma cell migration and invasion through activation of the extracellular signal-regulated kinase (ERK) kinase (MEK)/ERK signalling pathway.

**Conclusion:**

Ankyrin repeat-rich membrane spanning expression, conjunctly with tumour thickness or ulceration, may serve as a prognostic factor in patients with cutaneous melanoma.

Cutaneous malignant melanoma is a highly aggressive cancer with poor prognosis at advanced stage. It originates from malignant transformation of melanocytes or nevocytes. During tumour progression from radial growth phase to invasive vertical growth phase, melanoma cells acquire metastatic capability and are usually resistant to current chemotherapy (Miller and Mihm, 2006). Several histological factors have been shown to correlate with the survival of melanoma patients, among which Breslow tumour thickness is recognised as the most important prognostic parameter (Sober *et al*, 1979). Although patients with thin melanomas have an excellent prognosis following curative surgery, those patients with increased thickness of the lesions (>1.0 mm Breslow tumour thickness) are at risk for distant metastasis and poor survival (Balch *et al*, 2001, 2009).

Reflecting its ontogenic origin as neural crest cells, melanoma frequently re-expresses molecules functioning in neuronal development. For example, muscarinic acetylcholine receptors, neuron-specific enolase, and microtubule-associated protein 2 are overexpressed in malignant melanomas (Dhillon and Rode, 1982; Lammerding-Koppel *et al*, 1997; Soltani *et al*, 2005). We have demonstrated that ARMS (ankyrin repeat-rich membrane spanning), a neuron-enriched transmembrane molecule interacting with Trk, p75^NTR^, Eph, and protein kinase D (Iglesias *et al*, 2000; Kong *et al*, 2001; Chang *et al*, 2004), is overexpressed in cutaneous melanoma, while normal melanocytes and benign nevi reveal negative or weak expression of ARMS (Liao *et al*, 2007). Expression of ARMS in melanoma cells can confer resistance to UVB-induced apoptosis through activating the MEK/extracellular signal-regulated kinase (ERK) signalling pathway and thus facilitates neoplastic transformation (Liao *et al*, 2007). In this study, we investigated the role of ARMS in the malignant progression of cutaneous melanoma. We analysed and statistically correlated the immunointensity of ARMS in archived tissues with the clinicopathological characteristics of patients with cutaneous melanoma. Besides, statistical analysis was performed to correlate the expression level of ARMS with the patient overall survival. We further used RNA interference (RNAi) to knock down the expression of ARMS in mouse and human melanoma cell lines, and explored whether ARMS regulated the migratory/invasive ability of melanoma cells via *in vitro* and *in vivo* assays.

## Materials and methods

### Antibodies

Polyclonal anti-ARMS antibody was generated as described (Liao *et al*, 2007).

### Case selection

A total of 100 melanoma lesions were selected by searching the pathological records from 1997 to 2006 registered in National Taiwan University Hospital (NTUH). Tissue blocks were derived from the archives of Department of Pathology, NTUH, following the guidelines set forth by Tissue Committee at NTUH. Selected demographic information, including patient age, gender, tumour location, tumour subtype, and outcome of survival was retrieved from the hospital cancer registry.

### Tissue microarray and immunohistochemistry

Tissue microarrays were constructed from archival formalin-fixed, paraffin-embedded surgical specimens registered at Department of Pathology, NTUH. Sampling of tumour regions was based on visual alignment with the corresponding haematoxylin–eosin-stained sections. Tissue cores were placed into a recipient paraffin block with a tissue microarrayer (Manual Tissue Arrayer 1; Beecher Instruments, Inc., Sun Prairie, WI, USA), and 5-*μ*m sections of the resulting microarray block were used for immunohistochemistry. Immunohistochemistry for ARMS staining was performed as previously described (Liao *et al*, 2007). The sections were examined, and the staining intensity of ARMS was scored by two pathologists.

### Cell culture, RNAi, and selection of stable-transfected clones

Mouse B16-F0, -F1, -F10 and human A2058, A375 melanoma cell lines were from the American Type Tissue Culture Collection (ATCC; Manassas, VA, USA) and maintained in DMEM supplemented with 10% FBS, 4 mM L-glutamine, 100 *μ*g ml^–1^ streptomycin, and 100 U ml^–1^ penicillin. The siRNA expression vector pSUPER.neo+GFP (OligoEngine, Seattle, WA, USA) was used for expression of ARMS siRNA or control scrambled siRNA in B16-F10 cells. The specific sequence for paired RNA oligonucleotides contained nucleotides 4271–4289 for ARMS–RNAi-1 and 4840–4858 for ARMS–siRNA-2 in mouse ARMS (accession number: AK122478). Transfection and selection of ARMS knockdown stable cell clones were performed as previously described (Liao *et al*, 2007). The RNAi rescue experiment was performed by transfecting the ARMS–RNAi-2/B16-F10 stable clone with the ARMS–pcDNA3.1C-Myc 4849T → C expression mutant as previously described (Liao *et al*, 2007). Lentivirus carrying ARMS-specific and scrambled small interfering RNA were purchased from the National RNAi Core Facility of Academia Sinica (Taipei, Taiwan). The sequences used for ARMS targeting was 5′-GCACTGATTGTGGCAGTGAAA-3′. To generate recombinant lentivirus, 293FT cells were co-transfected with the package, envelop, and siRNA-expressing constructs. The virus-containing supernatant was harvested and then used to infect A2058 and A375 human melanoma cells. The infected cells were selected with puromycin 2 *μ*g ml^–1^ and the stable clones with the highest knockdown efficiency were used for subsequent experiments.

### Invasion assays

The invasiveness of melanoma cells were assayed using Boyden chambers containing polycarbonate filters (Transwell 24, 8-*μ*m pore; Costar, Cambridge, MA, USA). Filters were coated with 2 mg ml^–1^ Matrigel (Becton Dickinson, Heidelberg, Germany) at 37 °C overnight. Cells were harvested, resuspended in medium, and placed in the upper wells at a density of 5 × 10^4^ in 100 *μ*l DMEM. In some cases, 10 *μ*M PD98059 (Calbiochem, La Jolla, CA, USA) or dimethyl sulfoxide (DMSO; 0.02%) was added to the upper and lower chambers. After incubation for 48 h at 37 °C, membrane toward upper chamber is scraped by cotton swabs. The cells adhering to the lower surface were pre-fixed with 4% paraformaldehyde, stained with crystal violet, and counted by microscope observation. For each membrane, the mean number of cells of five randomly selected fields (100 × ) in the centre of the filter was obtained. The experiments were performed in triplicate.

### Scratch wound assay

Melanoma cells were seeded in high density (5 × 10^5^) into 35 mm dishes. Scratch wounds were made by creating three linear denuded regions using a pipette tip per dish. The cells were washed twice with medium before their incubation. In some cases, 10 *μ*M PD98059 or DMSO 0.02% was added in the medium. Six fields per dish were photographed at 0 and 17 h. Migration into the wounds was documented and measured by MetaMorph7.6 Software (Molecular Devises Inc., Sunnyvale, CA, USA). Each analysis was performed in triplicate.

### *In vivo* metastasis assay

Control and ARMS–RNAi stable B16-F10 cell lines were individually injected into C57BL/6J mice via the lateral tail vein (5 × 10^5^ cells per mouse, *n*=5 in each group). Mice were killed 10 days after intravenous injection. The lungs were fixed, photographed, and the number of visible black nodules on the lung surface was counted with a dissecting microscope. The lung tissue was then embedded in paraffin and sectioned at three different levels; the sections were stained with haematoxylin and eosin for light microscopy.

### Statistical analysis

The correlation between the intensity of ARMS expression and the clinicopathological parameters was evaluated by two-sided Fisher's exact test. The impact of immunointensity of ARMS, tumour thickness, ulceration, tumour location, age, and sex on patient overall survival was assessed by the Kaplan–Meier method, and differences between groups were analysed by the log-rank test. Multivariate Cox proportional hazard regression analysis was used to assess independent variables. *P*<0.05 were considered statistically significant. Statistics were performed with the SPSS 12.0 software (SPSS Inc., Chicago, IL, USA) and calculations on patient survival were performed using the GraphPad Prism software (GraphPad Software Inc., San Diego, CA, USA).

## Results

### Overexpression of ARMS in primary and metastatic melanoma with enhanced immunoreactivity at tumour invasive fronts

We examined ARMS expression in tumours of different tissue type by immunohistochemistry on tumour tissue arrays. In this screening, ARMS was specifically expressed in tumours of neuroepithelium origin, which included pheochromocytoma, paraganglioma, central neurocytoma, and malignant melanoma (Supplementary Figure S1A). Other epithelial-derived tumours had barely or none detectable ARMS, which included 13 breast cancers (6 ductal, 2 lobular, 3 medullary, 1 papillary, and 1 metaplastic carcinoma), 12 lung cancers (5 squamous cell carcinoma and 7 adenocarcinoma), 6 gastric adenocarcinoma, 7 colon adenocarcinoma, 5 pancreatic adenocarcinoma, and 5 hepatocellular carcinoma (Supplementary Figures S1B–F). For skin cancers, expression of ARMS was specific to malignant melanoma in that non-melanocytic skin cancers such as basal cell carcinoma (*n*=5), squamous cell carcinoma (*n*=5), and extramammary Paget's disease (*n*=3) did not express ARMS (Supplementary Figures S1G–I). The results indicate that ARMS is specifically expressed in tumours of neuroectodermal origin.

**Figure 1:**
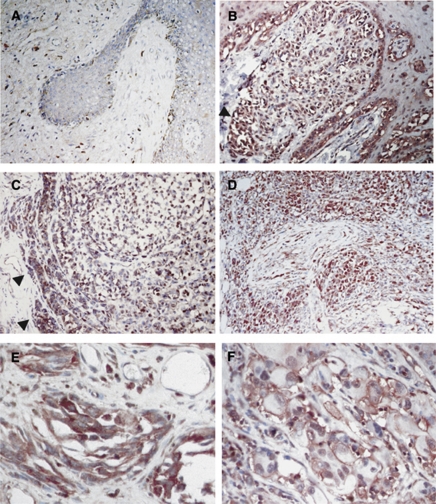
Immunohistochemical analysis of ARMS in cutaneous melanoma. Representative immunohistochemistry of ARMS in melanoma *in situ* and melanoma at vertical growth phase. Ankyrin repeat-rich membrane spanning staining was negative in melanoma *in situ* ( × 200, **A**). Stronger immunointensity of ARMS was shown at the invasive front ( × 200, **B** and **C**, arrow heads) and in tumour cells invading the nerve in the dermis at vertical growth phase ( × 100, **D**). Either cytoplasmic granular ( × 400, **E**) or membranous ( × 400, **F**) distribution pattern of ARMS was observed in cutaneous melanoma.

On the basis of the results from the immunohistochemistry screening of ARMS in tumour tissue arrays, we collected 100 cases of primary cutaneous melanoma and metastatic melanoma for further study. In accordance with our previous report (Liao *et al*, 2007), positive immunoexpression of ARMS was detected in 94.4% of cases with primary malignant melanoma as well as in 87% of cases with metastatic melanomas (Table 1). For primary melanoma, those ARMS-negative cases were all *in situ* lesions (Figure 1A). When the immunointensity of ARMS was further graded into weak, moderate, and strong intensity, stronger immunoreactivity of ARMS was found at the invasive front of tumour nests facing toward the dermis (Figures 1B and C, arrowheads) and in tumour cells invading the nerves (Figure 1D). These observations suggest that ARMS might be involved in the processes mediating local tumour invasiveness. However, when the immunointensity of ARMS was compared between metastatic melanoma and primary melanoma, no statistical significance was observed (Fisher’s exact test, *P*=0.58; Table 1). Immunohistochemistry of ARMS demonstrated two staining patterns: cytoplasmic granular and membranous distribution (Figures 1E and F). Both patterns were seen in primary cutaneous and metastatic melanoma with a similar percentage.

### The immunointensity of ARMS correlates with tumour thickness in cutaneous melanoma

The clinical outcome of patients with cutaneous melanoma is influenced by several factors including age, gender, ulceration, tumour subtype, and in particular, tumour thickness (Balch *et al*, 2001). To examine whether ARMS expression in cutaneous melanoma was associated with any clinicopathological parameter, we correlated each demographic characteristic with the immunointensity of ARMS among the 54 patients with primary cutaneous melanoma (Table 2). In this study group, 42 cases (77.8%) were diagnosed as acral lentiginous melanoma, 5 cases (9.3%) as nodular melanoma, and 7 cases as superficial spreading melanoma (13%). There were 26 men and 28 women included in this study group, and their age ranged from 28 to 88 years old (the median, 64 years). We used Breslow tumour thickness to evaluate local invasion of melanoma. By this way, 15 cases (27.8%) were classified as the subgroup with lesion ⩽1.0 mm, and the remaining 39 cases (72.2%) were within the subgroup with lesion >1.0 mm.

Analysis of ARMS expression and the clinical parameters including age, gender, ulceration, and tumour subtypes did not reveal any significant association (Table 2). However, when the immunointensity of ARMS was compared in terms of Breslow thickness, melanomas with tumour thickness >1.0 mm tended to have stronger ARMS immunoreactivity than lesions of melanoma *in situ*. Positive ARMS staining was detected in all cases of melanoma with thickness >1.0 mm, of which 23.1% were strongly stained. Whereas for thin melanomas (thickness <1.0 mm), 20% was nonreactive to ARMS, 80% showed weak-to-moderate ARMS immunointensity, and none of them showed strong immunoreactivity toward ARMS (Fisher's exact test, *P*=0.002; Table 2). When the immunointensity of ARMS was compared between tumours with and without nodal/distant metastasis, 23.3% of tumours with nodal metastasis showed strong ARMS expression, compared with 8.3% of those without nodal metastasis (Fisher's exact test, *P*=0.026; Table 2). Combined all together, it suggests that ARMS expression is strongly upregulated in the cutaneous melanomas with tumour thickness >1.0 mm, and overexpression of ARMS may be related to local dermal invasion and distant nodal metastasis.

### Prognostic relevance of ARMS expression

**Figure 2:**
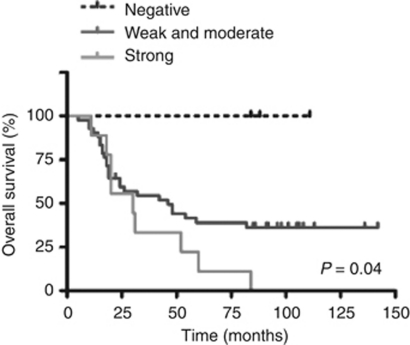
Kaplan–Meier survival analysis between ARMS expression and the overall survival in melanoma patients. Patients with negative-, weak-, or moderate-ARMS expression have a significantly better overall survival than those with strong-ARMS expression (*P*<0.05, log-rank test).

To evaluate whether the expression of ARMS in primary cutaneous melanomas was related to patient outcome, a Kaplan–Meier survival curve was constructed. The influence of tumour thickness and ulceration, two well-recognised prognostic factors on overall survival for cutaneous melanoma (Balch *et al*, 2009), was also assessed in this study group as a method standard and a comparison. Our analysis showed that both Breslow thickness (log-rank test, *P*=0.001) and tumour ulceration (log-rank test, *P*=0.007) were significant prognostic factors in our patients with primary cutaneous melanoma. Importantly, a significant correlation between the immunointensity of ARMS and the patient overall survival was shown (log-rank test, *P*=0.04; Figure 2). Shorter median survival time in melanoma patients with stronger ARMS expression (30 months) was revealed in comparison with the patients with weak-to-moderate ARMS immunointensity (42 months). Therefore, the expression level of ARMS may serve as a molecular predictor for the prognosis of patients with primary cutaneous melanoma.

To investigate whether the immunointensity of ARMS was an independent prognostic marker for melanoma, we applied a multivariate Cox's proportional hazards model for the assessment of the overall survival. The variables taken into consideration included age, sex, tumour thickness, ulceration, tumour location, and intensity of ARMS staining. Such analysis demonstrated that only tumour thickness retained its significant predictive value for the overall survival (*P*=0.03, Supplementary Table 1). While comparing patients with strong immunointensity to those with negative, weak, or moderate ARMS staining, the hazard ratio was 1.28 (95% confidence interval 0.446–3.69, *P*=0.64) without reaching statistical significance (Supplementary Table 1).

### ARMS-knockdown diminishes migratory and invasive abilities of melanoma cells *in vitro* and *in vivo* through downregulation of MEK/ERK activity

**Figure 3:**
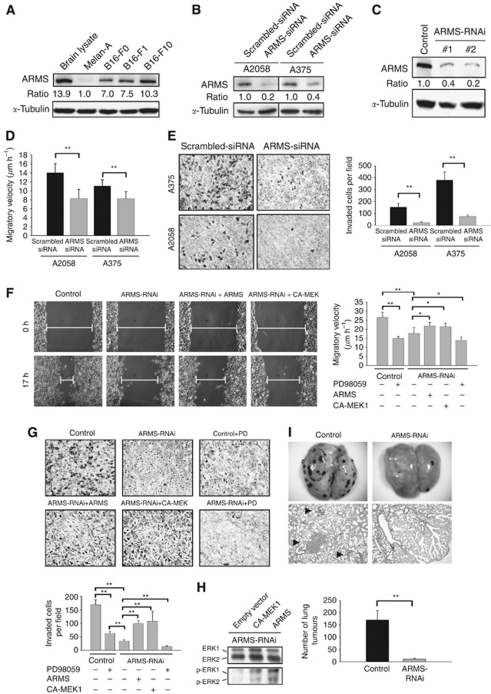
Depletion of ARMS deteriorates migratory and invasive abilities in melanoma cells via MEK/ERK signalling. (**A**) Western blot and quantification of ARMS protein levels in mouse melanoma B16-F0, -F1, and -F10 cell lines as normalised to *α*-tubulin using densiometry showed increased expression of ARMS in B16-F10 cells, the most aggressive melanoma cell line among them. Melan-A cells were given a value of 1 for comparison. Mouse brain lysate was used as a positive control. (**B**) Western blotting showed effective ARMS knockdown by siRNA in human A2058 and A375 melanoma cells. (**C**) Effective RNAi of endogenous ARMS in two independent mouse B16-F10 ARMS–RNAi stable cell clones as compared with their controls (scrambled). Levels of *α*-tubulin were shown as a loading control. (**D** and **E**) ARMS knockdown in human A2058 and A375 melanoma cells resulted in a reduction of migration velocity (bar graph, means±s.d.) in a wound-scratch assay (**D**) and decreased the invasive ability of the cells in a Boyden chamber invasion assay (**E**). Representative photomicrographs with the cresyl violet stain and bar graph (means±s.d.) demonstrated the status of cellular invasion between the control and ARMS–RNAi cells in a Boyden chamber invasion assay (magnification × 200). The experiments were performed in triplicate. ^**^P<0.01, Student's *t-*test. (**F** and **G**) ARMS silencing leaded to a significant reduction of migratory and invasive ability of B16-F10 cells via MEK/ERK signalling. Forced expression of RNAi-resistant ARMS or constitutively active MEK-1 (CA-MEK1) into B16-F10 melanoma cells with ARMS-knockdown resulted in a significant increase in cellular migratory ability (**F**, representative images and bar graph) and invasive potential in a Boyden chamber invasion assay (**G**, representative images and bar graph). Inhibition of MEK/ERK by PD98059 treatment in control and ARMS–RNAi cells compromised cell migration (**F**, bar graph) and invasion (**G**, images and bar graph). ^*^P<0.05; ^**^P<0.01; Student's *t-*test. (**H**) Overexpression of RNAi-resistant ARMS or CA-MEK1 in ARMS–RNAi cells resulted in ERK1/2 phosphorylation in melanoma cells compared with ARMS–RNAi cells. (**I**) The number of lung metastatic melanoma nodules was significantly decreased in mice receiving intravenous injection of ARMS–RNAi melanoma cells compared with that of control mice. Tumour nodules were shown in representative grossly dissected lungs (upper panels) and mouse lung tissue sections stained with haematoxylin and eosin (lower panels, × 200) in each group. ^**^P<0.01, Student's *t-*test.

To investigate the role of ARMS in tumour invasion and migration, we first examined the expression of ARMS in a panel of B16 melanoma cell lines with differential migratory ability. Western blot analysis revealed a trend of progressively increased ARMS expression associated with enhanced cell migratory ability. As shown in Figure 3A, ARMS protein was expressed at low levels in the benign mouse melanocyte cell line Melan-A (set as 1 for comparison), and was moderately increased in the less metastatic B16-F0 cells and B16-F1 cells (7-fold and 7.5-fold increase, respectively). By contrast, in highly metastatic B16-F10 melanoma cells, high levels of ARMS expression were observed (10.3-fold increase). When immunofluorescence was performed in human melanoma cell lines, colocalisation of ARMS with actin and relative enhancement of ARMS in invading filopodia or lamellipodia were observed (Supplementary Figure S2). Taken together, these results suggest that ARMS may be involved in melanoma progression by regulating cell migration and invasion.

To explore the cause–effect relationship between ARMS expression and melanoma aggressiveness, we used RNAi to knockdown ARMS expression in three metastatic melanoma cell lines with abundant ARMS expression (human A2058, human A375, and mouse B16-F10 cells). Expression of ARMS in human A2058 and A375 cells was silenced to the level around 20 and 40%, respectively, of that in control scrambled cells (Figure 3B). For mouse B16-F10 melanoma cells, two independent clones targeted by different nucleotide sequences (designated as ARMS–RNAi-1 and ARMS–RNAi-2) had ARMS expression to the level around 40 and 20% of that in control, respectively (Figure 3C). The proliferation rate of these constructed stable clones was not affected by ARMS silencing, as evidenced by Trypan blue exclusive assay (data not shown).

The migratory and invasive capacities of melanoma cells with or without ARMS–RNAi were assessed by *in vitro* wound scratch and chamber invasion assays. As shown in Figure 3D, ARMS silencing decreased migratory velocity in both A2058 and A375 cells. The mean migratory velocity in A2058 cells was 8.3±2.0 *μ*m h^–1^ for ARMS–RNAi *vs* 14.0±2.1 *μ*m h^–1^ for control cells, and in A375 cells was 8.2±1.6 *μ*m h^–1^ for ARMS–RNAi *vs* 11.1±1.4 *μ*m h^–1^ for control cells (Student's *t*-test, *P*<0.01). Knockdown of ARMS also decreased invasion capacity of A2058 (mean±s.d.; 154.0±31.4 cells per 100 × field in control cells *vs* 25.3±4.7 cells in ARMS–RNAi cells) and A375 cells (380.7±68.5 cells in control cells *vs* 76.2±12.5 cells in ARMS–RNAi cells) as evidenced by Boyden chamber invasion assay (Figure 3E; Student's *t-*test, *P*<0.01). Similarly, ARMS silencing in mouse B16-F10 melanoma cells significantly demised the migration potential of tumour cells in wound scratch assay (17.8±3.1 *μ*m h^–1^ for ARMS–RNAi *vs* 26.5±2.9 *μ*m h^–1^ for control) and led to a significant decrease in their invasive capability (169±17.6 cells per 100 × field in control cells *vs* 35±4.4 cells in ARMS–RNAi cells; Figure 3F; Student's *t-*test, *P*<0.01). Reintroduction of RNAi-resistant full-length ARMS into ARMS-knockdown cells significantly recovered the compromised migratory and invasive capability of melanoma cells resulting from ARMS depletion as evident by wound scratch (21.6±2.0 *μ*m h^–1^, 43.5% recovery compared with ARMS–RNAi cells; Figure 3F) and by the chamber invasion assay (99.7±10.5 cells per 100 × field, 49.3% recovery compared with ARMS–RNAi cells; Figure 3G). These results suggest that knockdown of ARMS significantly decreases the migratory and invasive potential of human A2058, A375, and murine B16-F10 melanoma cells.

Given that MEK/ERK molecules are downstream effectors of ARMS, we thus examined the role of MEK/ERK signalling in ARMS-mediated migration and invasion. First, we introduced constitutively active MEK1 (CA-MEK1) into ARMS-knockdown cells and observed that the compromised migratory and invasive capability of melanoma cells resulting from ARMS depletion was recovered (Figures 3F and G). Second, treatment of B16-F10 control cells with a MEK-specific inhibitor PD98059 resulted in a significant decrease in migratory and invasive ability similar to that in ARMS–RNAi cells (Figures 3F and G). Third, treatment of ARMS–RNAi cells with PD98059 further inhibited cell migration and invasion in comparison with vehicle-treated ARMS–RNAi cells (Figures 3F and G). Western blotting confirmed that both phospho-ERK1/2 were markedly upregulated by expression of CA-MEK1 and RNAi-resistant ARMS in the ARMS–RNAi cells that had decreased phosphorylated ERKs, while the expression levels of total ERK proteins were not altered (Figure 3H). Collectively, these results suggest that MEK/ERK signalling, which is downstream of ARMS regulates the migratory and invasive abilities in melanoma cells.

We then examined whether ARMS depletion interfered with the metastatic behaviour via murine lung metastasis assay. In all, 5 × 10^5^ control or ARMS–RNAi B16-F10 cells were injected into C57BL/6J mice via the tail veins, and the animals were assessed for lung metastases 10 days after injection (*n*=5 in each group). As revealed in Figure 3I, an average of 170±38.8 lung tumours developed in mice injected with control scrambled cells. By contrast, only 12±3.5 lung tumours were seen in mice injected with ARMS–RNAi melanoma cells (Student's *t-*test, *P*<0.01). Combined all together, the *in vitro* and *in vivo* data suggest that ARMS silencing deters melanoma cells from invasion and distant metastasis through downregulation of MEK/ERK signalling activity.

## Discussion

In this study, we demonstrate that ARMS is overexpressed in primary melanoma and in metastatic melanoma as well. The immunointensity of ARMS tends to be stronger in melanoma with tumour depth >1.0 mm, and is intensively localised at the invasive front of melanoma nests in the dermis. Besides, stronger ARMS expression is more frequently found in cases of cutaneous melanoma with lymph node or distant metastasis. Kaplan–Meier analysis further demonstrates that increased ARMS expression correlates negatively with the overall survival of melanoma patients. Therefore, ARMS may serve as a prognostic molecular marker in association with tumour thickness in cutaneous melanoma.

We further demonstrate that ARMS participates in the regulation of migratory/invasive ability of melanoma cells by *in vitro* and *in vivo* assays. We provide evidence that ARMS-mediated migratory and invasive abilities depend on the activated MEK/ERK signalling pathway, because constitutively active MEK reverts the compromised migratory and invasive activities caused by ARMS depletion and MEK1 inhibitor PD98059 leads to decreased migratory and invasive potential in melanoma cells. Therefore, activation of the ARMS/MEK/ERK signalling pathway is important for the acquisition of an invasion and metastatic phenotype in melanoma cells. As ARMS forms protein complex with Trk receptor tyrosine kinases or p75 neurotrophin receptor, and can transduce neurotrophin-mediated signalling that leads to ERK activation (Kong *et al*, 2001; Chang *et al*, 2004; Arevalo *et al*, 2004, 2006), it is interesting to identify that expression of neurotrophin, neurotrophin receptors, and phospho-ERK1/2 increases in a stage-specific trend during melanoma progression, just as ARMS does as shown in this study (Brocker *et al*, 1991; Easty *et al*, 1995; Walch *et al*, 1999; Innominato *et al*, 2001; Shonukan *et al*, 2003; Smalley, 2003; Zhuang *et al*, 2005). Similar to the distribution of ARMS at invading fronts of melanoma nests in this study, expression of phospho-ERK and p75NTR is more intense towards the deep tumour margins of thick melanoma (Shonukan *et al*, 2003; Zhuang *et al*, 2005). Also in accordance with the positive correlation between ARMS expression and the overall survival in patients with cutaneous melanoma, statistical analysis also shows a worse overall survival for phospho-ERK expression in patients with melanoma over 1 mm in thickness (Zhuang *et al*, 2005).

It is presently unclear how ARMS regulates migration and invasion in melanoma cells. The colocalisation of ARMS with F-actin on the cell cortex and cell projections in melanoma cells suggests that actin reorganisation could be modulated by ARMS to affect cell motility. Noticeably, it has been reported that p75NTR, an interacting receptor with ARMS, can mediate neurotrophin-dependent melanoma cell migration through specifically interacting with actin-bundling protein fascin (Shonukan *et al*, 2003). A recent study also reveals that ARMS is concentrated on filopodia and lamellipodia in developing neurons to regulate neuronal branching and growth (Higuero *et al*, 2010). We thus speculate that ARMS might have a role in the regulation of cytoskeleton and hence affect cell motility in both physiological and pathological processes, which definitely needs further experiments to prove the hypothesis.

## Figures and Tables

**Table 1:** ARMS expression in primary cutaneous melanoma and metastatic melanoma (*n*=100)

	**ARMS immunostain intensity**					
**Lesion**	***n***	**Negative**	**Weak**	**Moderate**	**Strong**	***P*-value** a
Primary melanoma	54	3 (5.6%)	20 (37.0%)	21 (38.9%)	10 (18.5%)	
Metastatic melanoma	46	6 (13.0%)	17 (37.0%)	14 (30.4%)	9 (19.6%)	0.58

Abbreviation: ARMS=ankyrin repeat-rich membrane spanning.

a *χ*^2^-test.

**Table 2:** Statistical correlations between the immunointensity of ARMS and each clinicopathological characteristic in 54 patients with primary cutaneous melanoma

	**Intensity of ARMS staining**					
	**Negative**	**Weak**	**Moderate**	**Strong**	**Total**	***P*-value** a
*Age*						
⩽64	2 (7.4%)	11 (40.8%)	10 (37.0%)	4 (14.8%)	27	0.86
>64	1 (3.7%)	9 (33.3%)	12 (44.4%)	5 (18.5%)	27	
						
*Sex*						
Male	2 (7.7%)	10 (38.5%)	8 (30.8%)	6 (23.1%)	26	0.098
Female	1 (3.6%)	10 (35.7%)	14 (50%)	3 (10.7%)	28	
						
*Tumour thickness (mm)*						
⩽1.0	3 (20.0%)	2 (13.3%)	10 (66.7%)	0 (0%)	15	0.002^b^
>1.0	0 (0.0%)	18 (46.1%)	12 (30.8%)	9 (23.1%)	39	
						
*Ulceration*						
Absent	3 (7.7%)	16 (41.0%)	17 (43.6%)	3 (7.7%)	39	0.053
Present	0 (0.0%)	4 (26.7%)	5 (33.3%)	6 (40.0%)	15	
						
*Lymph node invasion or distant metastasis*						
Negative	3 (12.5%)	6 (25.0%)	13 (54.2%)	2 (8.3%)	24	0.026^b^
Positive	0 (0.0%)	14 (46.7%)	9 (30.0%)	7 (23.3%)	29	
						
*Tumour subtype*						
ALM	3 (7.1%)	15 (35.7%)	18 (42.9%)	6 (14.3%)	42	0.44
NM	0 (0.0%)	3 (60.0%)	0 (0%)	2 (40.0%)	5	
SSM	0 (0.0%)	2 (28.6%)	4 (57.1%)	1 (14.3%)	7	

Abbreviations: ALM=acral lentiginous melanoma: ARMS=ankyrin repeat-rich membrane spanning; NM=nodular melanoma; SSM=superficial spreading melanoma.

a Fisher’s exact test;

b *P*<0.05.
