# Supplementary figures and images for: Upregulated ankyrin repeat-rich membrane spanning protein contributes to tumour progression in cutaneous melanoma
Source: Br J Cancer. 2011 Feb 22;104(6):982–8. doi: 10.1038/bjc.2011.18 (PMC3065267; doi:10.1038/bjc.2011.18)

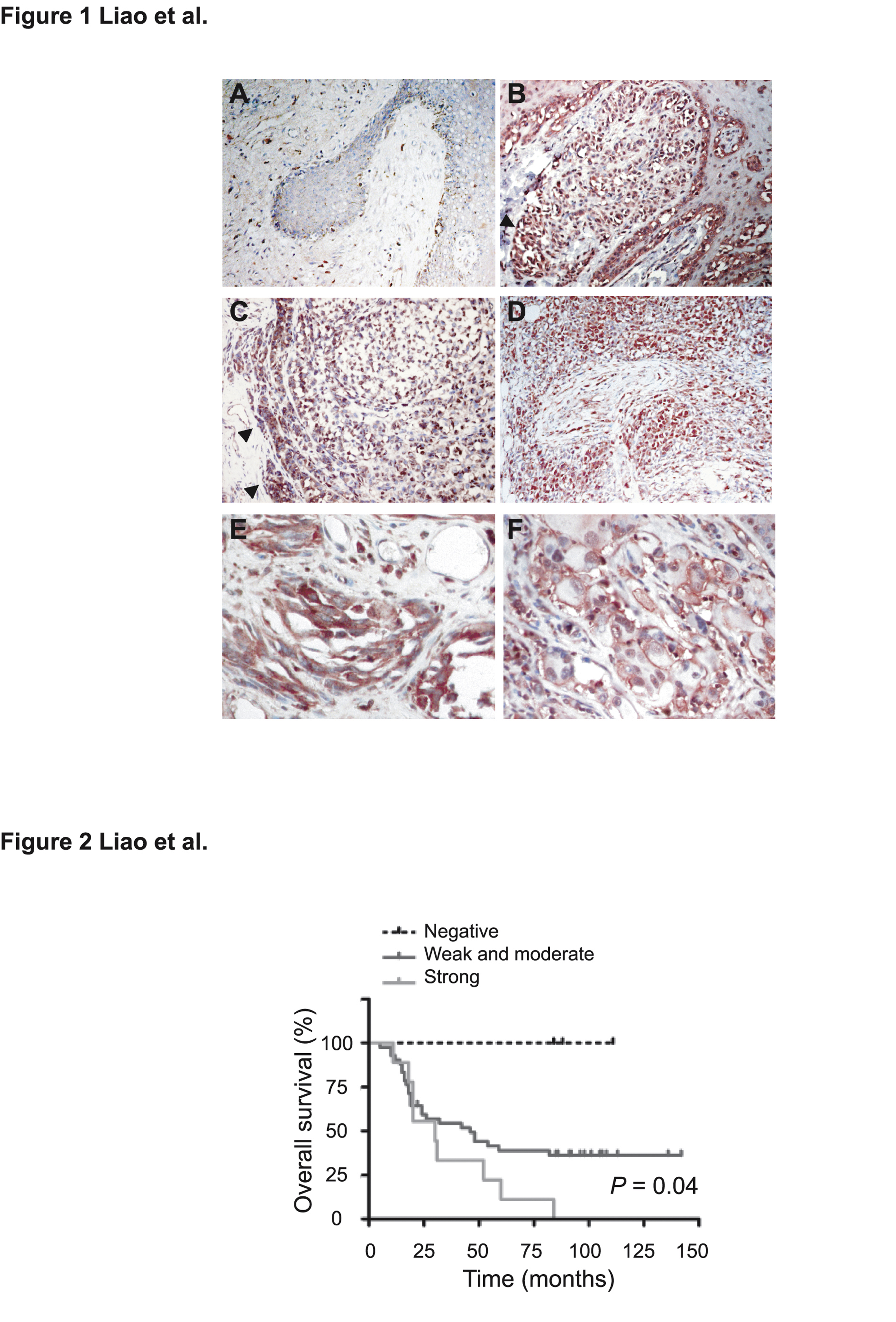

Supplement: Supplementary Figure S1 and S2 [file bjc201118x1.tif]
