# Supplementary material for: Upregulated ankyrin repeat-rich membrane spanning protein contributes to tumour progression in cutaneous melanoma
Source: Br J Cancer. 2011 Feb 22;104(6):982–8. doi: 10.1038/bjc.2011.18 (PMC3065267; doi:10.1038/bjc.2011.18)
Supplement: Supplementary Figure Legend [file bjc201118x2.doc]

**Supplemental Figure Legends**

**Figure S1** ARMS is specifically expressed in tumors of neuroendocrine origin. Representative photographs of the tissue arrays by ARMS immunohistochemistry. Strong immunoreactivity of ARMS was seen in tumors of neuroendocrine origin, such as central neurocytoma shown here (×400, **A**). Staining of ARMS revealed negative results in breast infiltrating ductal carcinoma (×200, **B**), lung adenocarcinoma (×200, **C**), gastric adenocarcinoma (×200, **D**), colon adenocarcinoma (×200, **E**), and hepatocellular carcinoma (×200, **F**). Non-melanocytic skin cancer including basal cell carcinoma (×400, **G**), squamous cell carcinoma (×400, **H**), and extramammary Paget’s disease (×200, **I**) also showed negative staining of ARMS.

**Figure S2** ARMS colocalizes with F-actin and is enriched at invasive fronts of melanoma cells. Human melanoma cell lines RPMI-7951 and SK-Mel-5 were stained with ARMS (green fluorescence) and phalloidin (red fluorescence). Merged pictures revealed colocalization of ARMS with F-actin in cellular cortex, lamellipodia, filopodia (magnified in middle panels), and cellular processes (lower panels, arrowheads).
