# Supplementary material for: Upregulated ankyrin repeat-rich membrane spanning protein contributes to tumour progression in cutaneous melanoma
Source: Br J Cancer. 2011 Feb 22;104(6):982–8. doi: 10.1038/bjc.2011.18 (PMC3065267; doi:10.1038/bjc.2011.18)
Supplement: Supplementary Table 1 [file bjc201118x3.doc]

**Supplemental Table**

**Table 1 Multivariate Cox regression analysis on the overall survival in 54 cases with primary melanomas**

| **Variable** | **Hazard ratio** | **95% CI** | ***P*** |
| --- | --- | --- | --- |
| “Strong” ARMS immunointensity | 1.28 | 0.446-3.69 | 0.6435 |
| Tumor thickness | 1.132 | 1.012-1.265 | 0.03* |
| Ulceration | 1.447 | 0.538-3.891 | 0.4638 |
| Non-acral located tumors | 1.722 | 0.753-3.938 | 0.1981 |
| Age | 0.998 | 0.971-1.025 | 0.8865 |
| Sex | 1.317 | 0.622-2.788 | 0.4713 |

CI, confidence interval; **P* < 0.05
